# Zinc Oxide Nanoparticles as Potential Delivery Carrier: Green Synthesis by *Aspergillus niger* Endophytic Fungus, Characterization, and In Vitro/In Vivo Antibacterial Activity

**DOI:** 10.3390/ph15091057

**Published:** 2022-08-26

**Authors:** Dalia H. Abdelkader, Walaa A. Negm, Engy Elekhnawy, Duaa Eliwa, Basmah N. Aldosari, Alanood S. Almurshedi

**Affiliations:** 1 Pharmaceutical Technology Department, Faculty of Pharmacy, Tanta University, Tanta 31527, Egypt; 2 Department of Pharmacognosy, Faculty of Pharmacy, Tanta University, Tanta 31527, Egypt; 3 Pharmaceutical Microbiology Department, Faculty of Pharmacy, Tanta University, Tanta 31527, Egypt; 4 Department of Pharmaceutics, College of Pharmacy, King Saud University, Riyadh 11451, Saudi Arabia

**Keywords:** *Aspergillus niger*, endophytes, nano-delivery system, systemic infection, *Staphylococcus aureus*, scanning/transmission electron microscopies

## Abstract

We aimed to synthesize zinc oxide nanoparticles (ZnO NPs) using the endophytic fungal extract of *Aspergillus niger*. The prepared ZnO NPs were characterized, and their in vitro and in vivo antibacterial activity was investigated. Isolated endophytic fungus identification was carried out using 18S rRNA. *A. niger* endophytic fungal extract was employed for the green synthesis of ZnO NPs. The in vitro antibacterial activity of the prepared ZnO NPs was elucidated against *Staphylococcus aureus* using the broth microdilution method and quantitative real-time polymerase chain reaction (qRT-PCR). Additionally, the in vivo antibacterial activity was elucidated using a systemic infection model in mice. The biosynthesized ZnO NPs showed a maximum optical density at 380 nm with characteristic peaks on the Fourier-transform infrared spectrum. The X-ray diffraction pattern was highly matched with a standard platform of zinc oxide crystals. Energy-dispersive X-ray analysis confirmed that the main composition of nanoparticles was zinc and oxygen atoms. Scanning and transmission electron microscopies showed spherical geometry with a smooth surface. Zeta potential measurements (26.6 ± 0.56 mV) verified the adequate stability of ZnO NPs. Minimum inhibitory concentrations of ZnO NPs against *S. aureus* isolates ranged from 8 to 128 µg/mL. Additionally, ZnO NPs revealed antibiofilm activity, resulting in the downregulation of the tested biofilm genes in 29.17% of *S. aureus* isolates. Regarding the in vivo experiment, ZnO NPs reduced congestion and fibrosis in liver and spleen tissues. They also improved liver function, increased the survival rate, and significantly decreased inflammatory markers (*p* < 0.05). ZnO NPs synthesized by *A. niger* endophytic fungus revealed a promising in vivo and in vitro antibacterial action against *S. aureus* isolates.

## 1. Introduction

Nanotechnology is a rapidly evolving scientific area with a broad range of implications for human life. Nanoparticles (NPs) are atom clusters with unique qualities such as their optical effect, quantum size effect, and surface effect with a size range of 1–100 nm [1,2]. These characteristics, which allow them to efficiently penetrate the cellular compartment of bacteria, plants, and animals, are determined by their shape, morphology, and size. Zinc oxide nanoparticles (ZnO NPs) are an essential candidate with various applications, including catalysis, electronics, medical diagnosis, antibacterial properties, and new activities such as anticoagulant, antidiabetic, and thrombolytic potential [2,3].

Different physical, chemical, and biological procedures produce ZnO NPs. Extreme circumstances, such as a large number of poisonous chemicals, high pressure, and high temperature, are required for physical or chemistry procedures, which affect the environment or necessitate complex equipment [4]. As a result, it is vital to design an environmentally safe approach for synthesizing ZnO NPs that uses gentle procedures and harmless ingredients. The use of biomaterials for ZnO NPs production has recently piqued researchers’ interest. Biomaterials include plant, fungi, bacteria, algae, arthropod metabolites, enzymes, animal, and agricultural waste materials [5,6]. 

The endophytic fungus lives in the hosts’ inner tissues and inside their plant cells, inflicting no visible symptoms or damage. According to studies, the host and endophytic fungi have a mutualistic relationship in which the host provides refuge and nutrients while the endophytes operate as chemical guardians. Endophytes in host plants have been shown to accelerate plant growth, increase disease resistance, and improve the plant’s ability to survive environmental stress, according to research so far [7,8]. Endophytic fungi are fascinating because they can produce secondary metabolites and have been effective in developing new drugs. Several investigations found that endophytic bacteria in plants have antibiotic action [9]. 

Endophytic fungi are often considered superior to other endophytic microorganisms because of their widespread and diversified nature; they produce a more significant number of secondary metabolites than other endophytic microorganisms. Natural product chemists are interested in endophytic fungus, searching for antibacterial or other active chemicals [10,11]. Endophytic microbes have been identified to play a significant role in generating pharmaceutically relevant chemicals in medicinal plants. According to the researchers, endophytic fungi isolated from medicinal plants produced substantial amounts of bactericidal, fungicidal, and cytotoxic compounds [12].

Herein, we aimed to synthesize ZnO NPs by *Aspergillus niger* endophytic fungus and characterize the produced nanoparticles using different techniques. This is in addition to the revealed in vitro and in vivo antibacterial activity against *Staphylococcus aureus* clinical isolates in an extensive way. Quantitative real-time polymerase chain reaction (qRT-PCR), histological studies, liver function tests, and enzyme linked immunosorbent assay (ELISA) were also carried out. 

## 2. Results

### 2.1. A. niger Endophytic Fungus

**Figure 1:**
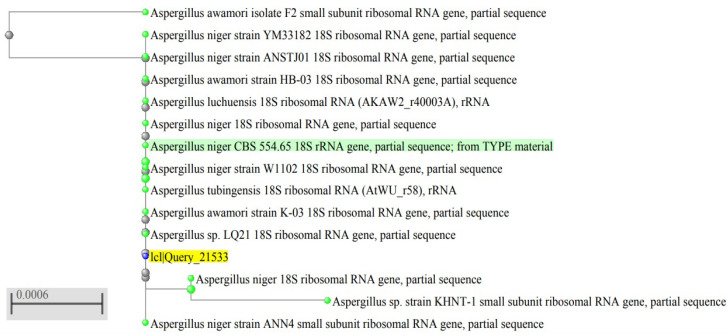
Phylogenetic tree of *A. niger* endophytic fungus (with yellow highlight) based on 18srRNA sequencing.

Based on the macroscopic and microscopic features of the isolated fungus, in addition to its molecular identification by 18S rRNA, as shown in Figure 1, the isolated fungus was identified as *A. niger*. DNA sequencing results were submitted to GenBank, and it had the accession number of ON100821, as shown in Table 1.

### 2.2. Characterization of Biosynthesized ZnO NPs from A. niger

**Figure 2:**
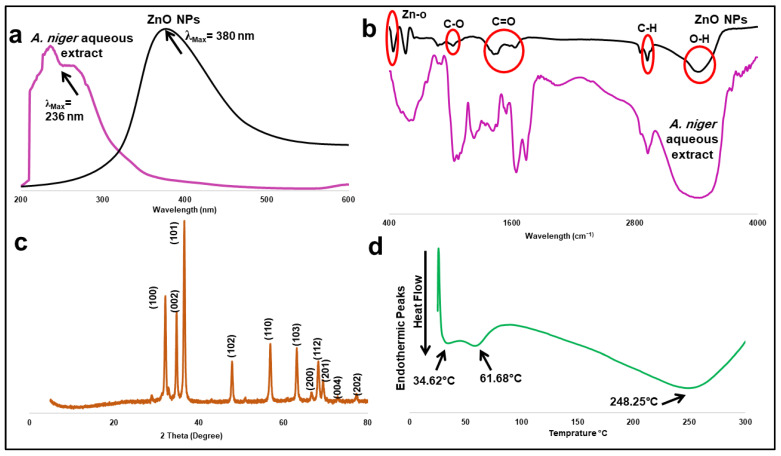
Different analytical spectra of ZnO NPs synthesized from *A. niger*. (**a**): UV spectrum, (**b**): FTIR bands, (**c**): XRD pattern, and (**d**): DSC curve.

The maximum absorption peak of ZnO NPs synthesized was 380 nm, demonstrating the synthesis of ZnO in a nano form (Figure 2a), while it was 236 nm in fungal aqueous extract. ZnO in a nano form has shorter absorption wavelengths than normal ZnO. The UV absorbance bands indicated the surface plasmon resonance (SPR) of the formed nanoparticles at the same absorbance with minimum shifts according to the shape and size of the nanoparticles. This finding is consistent with the previous results. The fungus-produced nanoparticles were white and suspended in distilled water. This color change in solution and a white ppt of ZnO NPs is further evidence of nano formation.

The presence of functional groups in the fungal extracts, which accounts for the bio-reduction and stabilization of the Zinc nanoparticles, was studied using the Fourier-transform infrared (FTIR) spectrum, obtained in the range of 400 cm^−1^ to 4000 cm^−1^ (Figure 2b). The strong broad peak at 3426.15 cm^−1^ signifies the presence of OH bonded stretch, signifying the presence of a carboxylic group. The peak at 2042.74 cm^−1^ indicates the presence of C–H bending stretch and in-plane deformation with aromatic ring stretching. Further, peaks at 1036.17 cm^−1^ correspond to C–N stretching, confirming the presence of amines, whereas the peaks at 630.87 cm^−1^ correspond to alkynes C–H bending planar vibrations [13]. 

Our findings are in line with Singh et al. [14], who confirmed that proteins bind to metallic nanoparticles through amines as well as electrostatic interactions. The comparative shifts (Figure 2b) in the FTIR spectra might be due to the interaction of the chemical moieties of the fungal extract with the metallic scaffold. Moreover, the carboxylic acids, phenolic acids, and amides in the enzymatic proteins of the fungal extract are probably essential for both the bio-reduction and stabilization of ZnO NPs. The results here agreed with previous reports of Aspergillus species mediated-nanoparticle synthesis, describing the involvement of fungal proteins in the bio-stabilization and the capping of the Zinc nanoparticles [15].

The FTIR spectrum (Figure 2b) showed characteristic bands of biosynthesized ZnO NPs, indicating that a metal-oxygen reaction occurred during preparation. A stretching vibrational band at 440.44 cm^−1^ ensured zinc and oxygen bonding [16,17]. Additionally, peaks at 1627.49, 1577.00, and 1424.13 cm^−1^ demonstrated bending vibrational bonds due to C=O [13]. A broad and strong stretching bond at 3420.03 cm^−1^ due to the hydroxyl group is clearly visible in Figure 2b [13,16,17]. At 2922.21 cm^−1^, a stretching band of C–H revealed alkane group formulation during the preparation of ZnO NPs [13]. Various active groups such as alcohol and/or ether might be structured at a wavelength of 1024.30 cm^−1^ due to the formation of C–O group [13,18]. 

The X-ray diffraction (XRD) pattern of ZnO NPs synthesized from the extract of *A. niger* is shown in Figure 2c, demonstrating peaks at 100, 002, 101, 102, 110, 103, 200,112, 201, 004 and 202 with 2 Theta values of 31.61, 35.03, 36.85, 48.13, 57.16, 63.41, 66.90, 68.49, 69.59, and 77.51, respectively [19] which is highly matched with the provided data of pure hexagonal Zinc oxide crystals available in our library supplied with an X-ray diffractometer [19,20]. Figure 2d shows the differential scanning calorimetry (DSC) curve of ZnO NPs, which displays three endothermic peaks at 34.62, 61.68, and 248.25 °C, respectively, which is similar to several previous findings [16,21].

**Figure 3:**
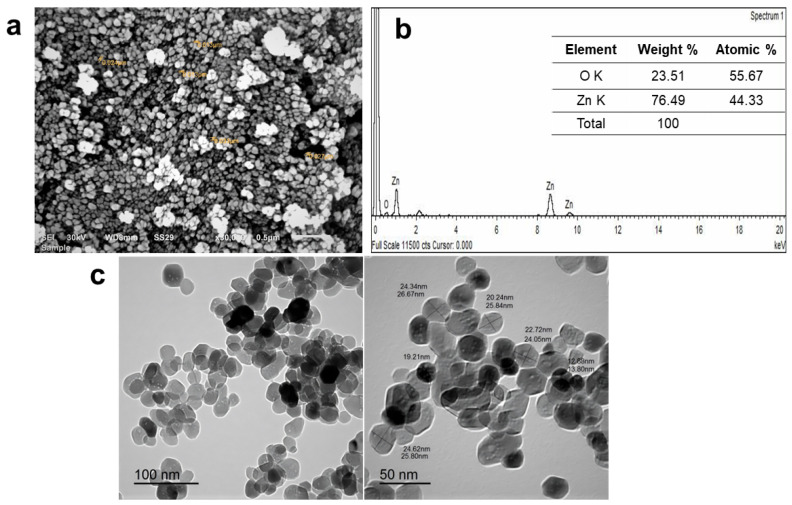
(**a**): SEM micrograph, (**b**): EDX spectrograph, and (**c**): TEM micrographs at different magnification powers of ZnO NPs synthesized from *A. niger.*

ZnO NPs showed spherical morphology (Figure 3a) with some hexagonal architecture displayed in the transmission electron microscope (TEM) micrographs (Figure 3c). After SEM analysis (Figure 3a), the average particle diameter of ZnO NPs is 31.75 ± 4.38 nm (data collected by calculating the average diameter of 50 individual nanoparticles using Image J software). In contrast, TEM imaging (Figure 3c) shows a mean diameter equal to 23.97 ± 2.29 nm, closely matching the readings of both scanning electron microscope (SEM) and TEM. The synthesis of ZnO NPs is obviously seen in the energy-dispersive X-ray (EDX) of NPs, as shown in Figure 3b. Oxygen’s atomic weight was 55.67%, while its current weight was 23.51%. At the same time, the atomic weight of Zinc was 44.33%, while the current weight was 76.49%. Peaks shown in the EDX spectrum (Figure 3b) demonstrate that the main constituents of synthesized NPs are Zinc and oxygen [16].

**Figure 4:**
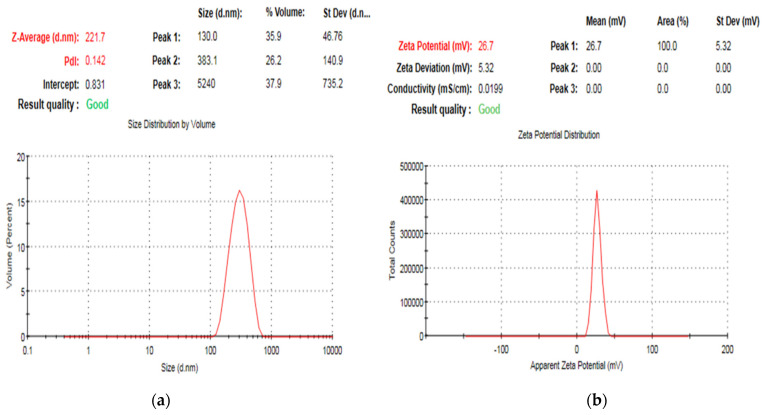
Representative photos showed measurements of (**a**): Particle size and PDI, (**b**): ζ-potential values displaying good quality results.

The ζ-potential and size distribution range of ZnO NPs were determined by the dynamic light scattering (DLS) technique (Figure 4). The average polydispersity index (PDI) value of ZnO NPs was 0.186 ± 0.05, which is considered an optimum value with adequate size distribution (Figure 4a). The hydrodynamic size of ZnO NPs was also evaluated using DLS (Figure 4a), showing an average diameter equal to 225.46 ± 3.27 nm. The value of ζ-potential critically determines the colloidal stability of ZnO nanosuspension after proper dilution with distilled water. Generally, the optimum value of ζ-potential ranges from +40 to −40 mV to ensure adequate dispersibility with no risk of aggregation or flocculation [22,23,24]. The surface charge of biosynthesized ZnO NPs from *A. niger* is equal to 26.6 ± 0.56 mV (Figure 4b).

### 2.3. In Vitro Antibacterial Activity

#### 2.3.1. Susceptibility to ZnO NPs

ZnO NPs produced an inhibition zone around the isolates by performing the agar well diffusion technique. This means that it has antibacterial activity against *S. aureus* isolates. Then, the minimum inhibitory concentration (MIC) values of ZnO NPs were detected by performing the broth microdilution method. As shown in Table S2, the MIC values of ZnO NPs ranged from 8 to 128 µg/mL. 

#### 2.3.2. Impact of ZnO NPs on Biofilm

Using the crystal violet assay, the phenotypic impact of ZnO NPs on biofilm formation was assessed. Interestingly, the ZnO NPs decreased the number of *S. aureus* isolates with a strong and moderate ability to form biofilm from 50% to 20.83%, as revealed in Table 2.

#### 2.3.3. qRT-PCR

**Figure 5:**
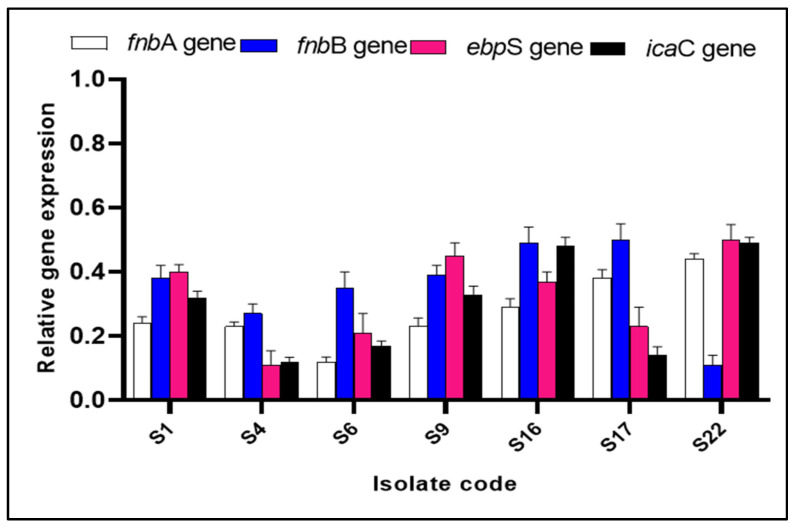
Bar chart showing the downregulation of the biofilm genes in the seven *S. aureus* isolates (S1, S4, S6, S9, S16, S17, S22) after treatment with ZnO NPs.

The impact of ZnO NPs on the expression of the biofilm genes was assessed using qRT-PCR. Figure 5 shows the downregulation of the biofilm genes induced by ZnO NPs in 29.17% of *S. aureus* isolates.

### 2.4. In Vivo Antibacterial Activity

#### 2.4.1. Bacterial Burden and Survival Curve

**Figure 6:**
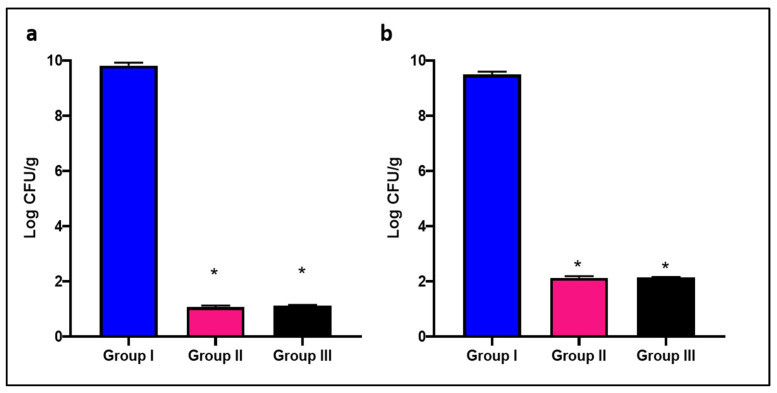
Bar chart showing the number of CFU/mL of *S. aureus* isolates in (**a**) liver and (**b**) spleen in the different experimental groups. The symbol (*) represents a significant decrease (*p* < 0.05) in comparison with group I.

**Figure 7:**
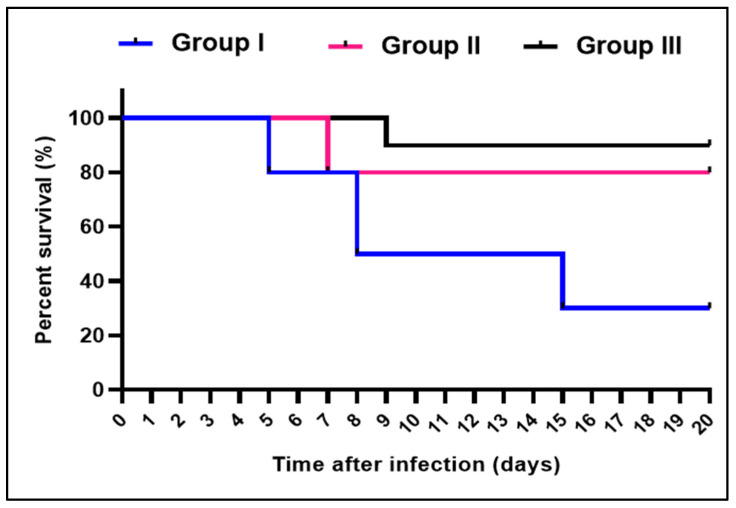
Kaplan–Meier survival curve of the experimental groups.

As revealed in Figure 6, ZnO NPs caused a significant decrease (*p* < 0.05) in the value of the colony forming unit (CFU/g) tissues of the liver and spleen. In addition, it increased the survival rate of the infected mice, as shown in Figure 7. Regarding group I, two mice died after five days, three died after eight days, two died after 15 days, and the rest remained alive. Only two mice died after one week, and one died after nine days in groups II and III, respectively. 

#### 2.4.2. Liver Function Tests

ZnO NPs caused a significant decline (*p* < 0.05) in alanine aminotransferase (ALT), aspartate aminotransferase (AST), and bilirubin levels. In addition, it caused a significant rise (*p* < 0.05) in the total proteins and albumin levels, as shown in Table 3. 

#### 2.4.3. Staining with Hematoxylin and Eosin (H&E) and Masson’s Trichrome Stain

**Figure 8:**
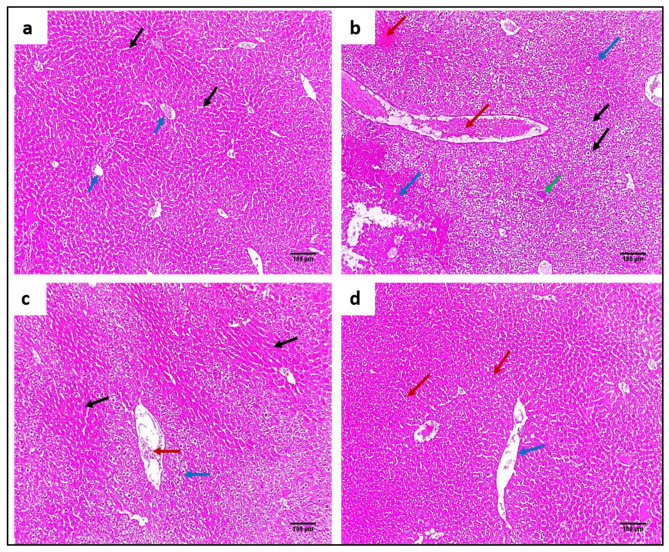
H&E micrographs of liver sections of (**a**) normal liver showing average-sized central veins (blue arrows) bounded by cords of hepatocytes separated by blood sinusoids (black arrows) (×100). (**b**) Group I showed congested vessels (red arrows) bounded by hepatocytes with hydropic degeneration (black arrows), apoptotic bodies (green arrow), and areas of necrosis (blue arrows) (×100). (**c**) Group II showed mild dilated-sized central veins (red arrow) bounded by cords of hepatocytes with hydropic degeneration (blue arrow), peripheral normal hepatocytes (black arrows), and no necrosis could be detected (×100). (**d**) Group III showed a mild dilated-sized central vein (blue arrow) surrounded by cords of average-sized hepatocytes (red arrows) (×100).

**Figure 9:**
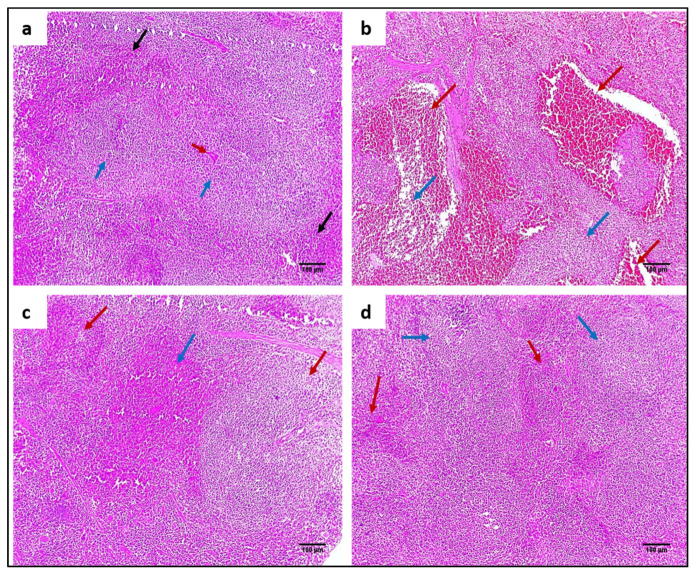
H&E micrographs of spleen sections of (**a**) normal spleen showing normal-sized white pulp (lymphoid follicles) (blue arrows) with central arteriole (red arrow) surrounded by average-sized red pulp (blood sinusoids) (black arrows) (×100). (**b**) Group I showed marked congestion exhibiting dilated congested red pulp and areas of hemorrhage (red arrows) with atrophic white pulp (blue arrows) (×100). (**c**) Group II showed mild congestion in the red pulp (blue arrow) with average-sized lymphoid follicles (red arrows) (×100). (**d**) Group III showed preserved white pulp (lymphoid follicles) (blue arrows) with average-sized red pulp (red arrows) (×100).

**Figure 10:**
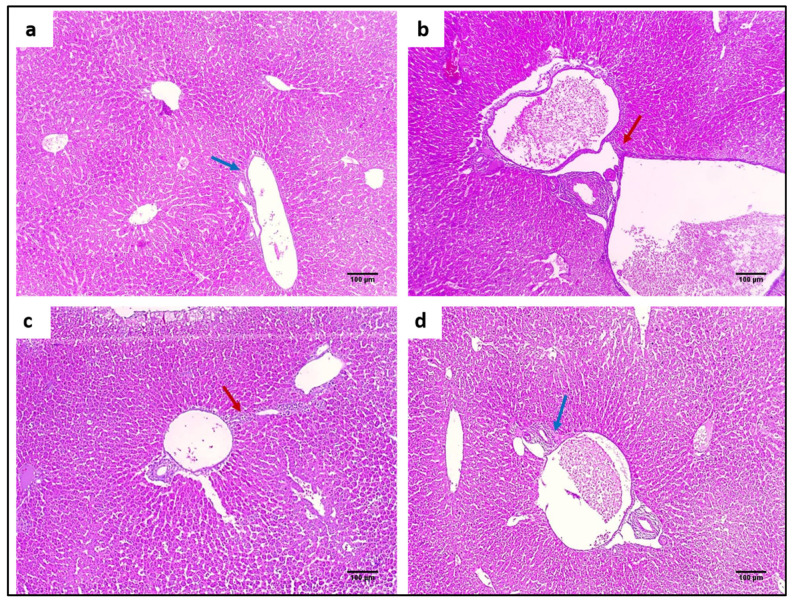
Masson’s trichrome-stained liver sections of (**a**) normal liver showing portal tract with a slight fibrous wall (blue arrow) and no fibrosis (×100). (**b**) Group I showed fibrous expansion of the portal areas with an occasional portal- portal (bridging) (red arrow) (×100). (**c**) Group II showed fibrous expansion of the portal areas with short fibrous septa (red arrow) (×100). (**d**) Group III showed a fibrous expansion of a few portal areas (blue arrow) (×100).

**Figure 11:**
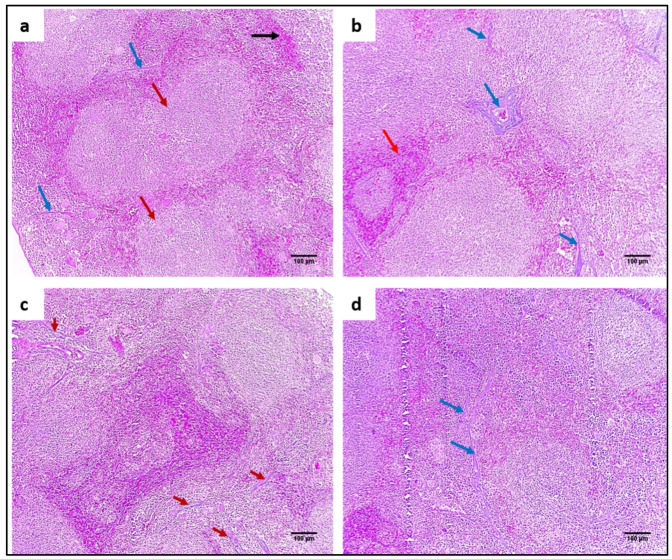
Masson’s trichrome stained spleen sections of (**a**) normal spleen showing splenic parenchyma formed of white (red arrows) and red pulps (black arrow). The stroma is represented by slight amounts of blue-stained collagen fibers (blue arrows) (×100). (**b**) Group I showed an area of congestion (red arrow) with multiple areas of blue-stained collagen fibres (blue arrows) (×100). (**c**) Group II showed a mild reduction of collagen fibres with remaining streaks of blue-stained collagen fibres (red arrows) (×100). (**d**) Group III showed a marked reduction of collagen fibres, and only the focal amount could be detected (blue arrows) (×100).

H&E staining was utilized to evaluate the impact of ZnO NPs on the tissues of the liver and spleen, as demonstrated in Figure 8 and Figure 9. In addition, Masson’s trichrome stain was used to stain collagen, as shown in Figure 10 and Figure 11.

#### 2.4.4. Detection of the Inflammation Markers by ELISA

ZnO NPs resulted in a significant decrease (*p* < 0.05) in interleukin (IL-6 and IL-1β) levels compared with the control group, as presented in Table 4. 

## 3. Discussion

Plant endophytes are a remarkable source for various bioactive compounds as they are capable of producing many metabolites such as those produced by the plant itself or even novel compounds. *A. niger* endophytic fungus possesses enormous amounts of secondary metabolites, which can play a role in the biogenic synthesis of ZnO NPs [25]. Thus, in the current study, after the isolation and identification of the endophytic fungus *A. niger*, we used it for the production of ZnO NPs to benefit from the advantages of the green synthesis of nanoparticles.

UV spectroscopy confirmed the formation of zinc oxide nanocrystals showing λ_Max_ consistent with several previous studies in the range of 330–390 nm (Figure 2a) [13,26]. Additionally, the FTIR spectrum displayed peaks that verified the formation of metal-oxygen bonding (Figure 2b) [13,16,17]. XRD spectrum showed no distinctive peak for any other impurities ensuring the purity of hexagonal nanocrystals composed only from Zinc oxide (Figure 2d) [26]. The three endothermic peaks of ZnO NPs shown on the DSC curve describe three subsequent steps that occurred during the formation of ZnO nanocrystals (Figure 2c). Firstly, the formulation of ZnO NPs from its precursor, followed by evaporation of the absorbed water molecules from the surface of the nanocomposite, and then the removal of any organic residues or impurities to maximize the degree of purity [21]. 

The positive value of ζ-potential lies within the range, indicating good colloidal stability of our nanosystem with optimal repulsion force. Additionally, the high positive charge of ZnO NPs synthesized from *A. niger* might be a combinatory charge of the main constituents of nanostructured particles plus the possible fungal metabolites adsorbed on the surface and eradicated during the green synthesis of ZnO NPs [13,26]. 

PDI readings ensured good homogeneity and all prepared ZnO NPs lay in a narrow range of size variation [27]. The DLS results showed a larger particle size of ZnO NPs (Figure 3a) compared with SEM (Figure 2a) and TEM (Figure 2c). This might be attributed to the different mechanisms used during measurements. SEM and TEM visualize the nanoparticles with their entire edges. The scale could record the exact diameter, whereas the DLS technique measured the hydrodynamic particle size via their scattering behavior against directed light beams to monitor the Brownian motion of the aqueous nanosuspension [13,16]. 

*S. aureus* is considered one of the most important pathogenic bacteria. This is attributed to its increased resistance to the existing antibiotics in addition to its virulence factors [28]. Therefore, there is a high need to discover new anti-infective therapeutic agents. 

Herein, ZnO NPs displayed antibacterial potential against the tested *S. aureus* isolates, and they had MIC values ranging from 8 to 128 µg/mL as found by performing the broth microdilution method. Nanoparticles are documented to have potent antimicrobial activity. Their very small particle size allows them to interact with the microbial surface. Then, they are able to enter the microbial cells and subsequently display their antimicrobial action [26]. 

Biofilms are communities of bacterial cells fixed in an extracellular matrix produced by cells. Biofilms can be found on different surfaces such as implanted medical devices. Unfortunately, the bacterial cells that form biofilms are 1000 times more resistant to current antibiotics than planktonic cells [29]. Thus, when biofilms are formed, they are usually difficult to eliminate. This would lead to the development of chronic and persistent infections. Biofilm-forming *S. aureus* can cause persistent infections which resist eradication using conventional antibiotic therapy [30]. 

Therefore, antibiofilm agents are important in treating bacterial infections, especially those caused by *S. aureus*. Here, we elucidated the antibiofilm activity of ZnO NPs using a crystal violet assay. Moreover, we explored its effect on the relative gene expression of the biofilm genes. Interestingly, ZnO NPs decreased the strong and moderate biofilm-forming cells from 50% to 20.83%. In addition, it resulted in a downregulation of the biofilm genes in 29.17% of *S. aureus* isolates. 

The in vivo model in the infected mice was utilized to mimic real infection in humans [31]. After the induction of systemic infection in mice, we studied the consequence of treating ZnO NPs on the histological characters as well as collagen in liver and spleen tissues. ZnO NPs reduced congestion, necrosis, degeneration, and fibrosis in liver and spleen tissues. In addition, the liver function was assessed, and it was found that ZnO NPs improved liver function and resulted in a higher survival rate than the control group. Additionally, inflammatory markers, including IL-6 and IL-1β, were evaluated in the different groups. During bacterial infection, the host cells produce proinflammatory cytokines that initiate the inflammatory process. We found that ZnO NPs resulted in a significant reduction (*p* < 0.05) in these cytokines.

## 4. Materials and Methods

### 4.1. Chemicals and Media

The utilized media in the current study were purchased from Oxoid, UK. Moreover, the chemicals were obtained from Merck, USA.

### 4.2. Endophytic Fungi

#### 4.2.1. Isolation

In this study, *A. niger* used for ZnO NPs synthesis was isolated from the healthy leaves of the plant *Acalypha hispida* Burm. f. collected from the farm of Faculty of Pharmacy, Tanta University, Tanta, Egypt, in September 2021. Leaf samples were thoroughly rinsed several times with sterile water and disinfected, from their superficial surfaces, using 70% ethanol for two minutes. Then, they were cut into relatively small pieces (2 × 2 cm) using a sterile dissection razor and placed on potato dextrose agar (PDA) plates with 250 mg/L amoxicillin to prevent contamination. The plates were left at room temperature for one to two weeks after sealing with parafilm until adequate fungus growth [32]. 

#### 4.2.2. Identification

Pure strains of *A. niger* were isolated on PDA plates [33] for molecular identification by 18S rRNA gene sequencing [33]. The whole genomic DNA of the fungal isolate was extracted using E.Z.N.A.^®^ High-Performance Fungal DNA Kits, Omega Bio-Tek (Suite, Norcross, Georgia), as described by the manufacturer. Then, DNA was amplified using the universal fungal primer, 18S rRNA [34]. Its sequence in the forward direction is 5’-CCTGGTTGATCCTGCCAGTA-3’, and in the reverse direction it is 5’-GCTTGATCCTTCTGCAGGTT-3’. The purified amplified products were sequenced at Macrogen Co., South Korea. The obtained sequences were placed in the Gene Bank (https://blast.ncbi.nlm.nih.gov/Blast.cgi, accessed on 1 June 2022). A BLAST search was performed to estimate the sequence homology of the fungal isolate with the closest related fungal strains. Finally, a phylogenetic tree was built using the MEGA 7.0 program.

### 4.3. Synthesis of ZnO NPs by A. niger

After the morphological and molecular identification of the isolate fungi, *A. niger* was grown in a sabouraud medium, which was inoculated with 1 × 10^6^ spores in 250 mL flasks. Flasks were incubated at 27 ± 2 °C in a rotary shaker (140 rpm) for 5 days. After fermentation, fungal biomass was harvested by filtration using Whatman filter paper and washed with sterile double distilled water to prevent contamination from the medium. Then, 20 g (wet weight) of biomass was placed in a flask containing 100 mL sterile distilled water and incubated at 27 ± 2 °C for 24 h. Then, the suspension was filtered [35].

A previously described *A. niger* filtrate was used to make ZnO NPs [36] with minor changes. To generate ZnO NPs, one gram of *A. niger* filtrate in 100 mL ethanol was added to 10 g zinc acetate dissolved in one liter of distilled water. This mixture was heated in a water bath for 30 min. Adding 0.1 M NaOH (just drops) increased the pH to 11, resulting in the development of ZnO NPs (white precipitate). The mixture was left at room temperature for one hour. After freeze-drying, the obtained ZnO NPs were centrifuged for 10 min at 4000 rpm, then rinsed at different times with distilled water and ethyl alcohol to yield about 850 mg of white powder of ZnO NPs.

### 4.4. In Vitro Characterization of A. niger Synthesized ZnO NPs

Various analytical methods have been employed to evaluate the physicochemical characteristics of ZnO NPs synthesized from *A. niger*. The reaction between the fungal filtrate and Zinc acetate during the preparation of ZnO NPs was ensured using the spectral techniques of UV-VIS and FTIR spectroscopy. XRD and EDX were used for crystal and elemental analysis, respectively. In contrast, SEM and TEM were utilized to visualize the surface and internal morphology of ZnO NPs. Additionally, the surface charge and hydrodynamic particle size of ZnO NPs were detected using DLS.

#### 4.4.1. UV Spectroscopy

The optical properties of *A. niger* synthesized ZnO NPs were examined using UV-visible spectrophotometry (Evolution 300 spectrophotometer, Thermo Scientific, Waltham, MA, USA). A sample (5 mg) of ZnO NPs was properly diluted and scanned at a wavelength range of 200–600 nm [13]. 

#### 4.4.2. FTIR Spectroscopy

The characteristic functional groups synthesized during the formulation of ZnO NPs were fully examined using FTIR spectrophotometer (Bruker Tensor, Billerica, MA, USA). A few milligrams of lyophilized ZnO NPs were physically mixed with potassium bromide and pressed under high pressure to form a compressed pellet ready for FTIR analysis at a wavelength ranging from 400 to 4000 cm^−1^ [24].

#### 4.4.3. XRD

The crystalline phase of ZnO NPs was identified using APD 2000 PRO X-Ray Diffractometer (GNR Instrumental Group, Novara, Italy) supplied with Cu Kα radiation at λ = 1.5406 nm. The instrument was operated at a current of 25 mA and a source power equal to 35 kV. The XRD pattern was recorded at 2 Theta, ranging from 20° to 80° with a step size of 0.02 [13].

#### 4.4.4. DSC

The thermal peaks of ZnO NPs were detected using DISCOVERY DSC25, WatersTM, TA instruments, New Castle, DE, USA. The instrument was set at heating and nitrogen purge rates equal to 10 °C/min, and 40 mL/min, respectively. The temperature range of 20–300 °C was used for scanning [23]. 

#### 4.4.5. Scanning and Transmission Electron Microscopies

The external surface geometry of ZnO NPs was inspected using a scanning electron microscope (SEM) (JSM-6510LV microscope, JEOL, Tokyo, Japan). Sputter coater SPI-MODULE TM (SPI, West Chester, PA, USA) was used in the gold coating of ZnO NPs. The elemental status of ZnO NPs was analyzed using EDX via an analysis X-ray unit (Oxford X-Max 20, Ontario, Canada) attached to a scanning electron microscope [34]. 

After the proper dilution of ZnO NPs, droplets of ZnO NPs suspension were fixed on the carbon grid and left for 10 min until drying prior to examination under a transmission electron microscope (TEM, JEM-2100 Electron Microscope, JEOL Ltd., Tokyo, Japan) [13].

#### 4.4.6. Particle Size, PDI, and Zeta Potential

The average particle diameter, size distribution range, and surface charge of ZnO NPs synthesized from *A. niger* were analyzed. Malvern Zetasizer Nano-zs90 (Malvern Instruments Ltd., Worcestershire, UK) was used to assess particle size (nm), PDI, and zeta potential (mV). For precise analysis, ZnO NPs samples were appropriately diluted [13].

### 4.5. In Vitro Antibacterial Activity

#### 4.5.1. Bacteria

Twenty-four *S. aureus* isolates were acquired from the department of pharmaceutical microbiology, Tanta University, Tanta, Egypt. *Staphylococcus aureus* (ATCC 29231) was used as a reference isolate.

#### 4.5.2. Susceptibility Testing and Determination of the MICs

The agar well diffusion technique was employed in the current study to elucidate the antibacterial potential of ZnO NPs against *S. aureus* isolates [24]. After the inoculation of the tested bacteria into Mueller–Hinton agar plates by sterile swabs, three wells were made using a sterile cork borer. Then, vancomycin (positive control), sterile water (negative control), and ZnO NPs were placed into these three wells. The plates were incubated overnight at 37 °C, and the produced inhibition zones were inspected [37]. 

MIC values of ZnO NPs were detected using the resazurin broth microdilution method in 96 well plates [38]. The solution containing ZnO NPs (1024 μg/mL) was serially diluted (twofold) in the wells using Mueller–Hinton broth (MHB); then, the bacterial suspensions were added (10^7^ CFU/mL). After overnight incubation at 37 °C, 20 μL of resazurin solution was added to each well and left for one hour at 37 °C. Color variations were detected to determine MIC values [39,40].

#### 4.5.3. Antibiofilm Activity

The antibiofilm activity was evaluated (at 0.5 MIC values) using a crystal violet assay. An ELISA reader determined the values of the optical density (OD) at 490 nm. The cut-off OD (ODc) was calculated by adding the mean OD to three standard deviations (SD) of the negative control [41]. *S. aureus* isolates were classified into the following four classes: non-biofilm development (ODc < OD < 2 ODc); weak biofilm development (2ODc < OD < 4 ODc); moderate biofilm development (4ODc < OD < 6 ODc); and strong biofilm development (6 ODc < OD) [37,42].

#### 4.5.4. Effect on the Gene Expression of the Biofilm Genes

The effect of ZnO NPs on the biofilm-related genes (*fnb*A, *fnb*B, *ebp*S, *ica*C) [43] was determined by qRT-PCR, and the utilized housekeeping gene was 16S rRNA. The sequences of the primers are presented in Table S1. After extracting total RNA with the GeneJET RNA purification kit (Thermo Scientific, Waltham, MA, USA) described by the manufacturer, cDNA was formed using a power cDNA synthesis kit (iNtRON Biotechnology, Seongnam, Korea). Then, cDNA was amplified by Power SYBR^®^ Green master mix (Thermo Scientific, Waltham, MA, USA) in a Rotor-Gene Q 5plex machine (Qiagen, Hilden, Germany). We used the 2^−ΔΔCt^ method for the calculation of the relative gene expression. The expression of the genes in the isolates before treatment was 1 [44].

### 4.6. In Vivo Antibacterial Activity

#### 4.6.1. Animals

Thirty adult male Swiss albino mice, with weights ranging from 22 to 25 gm, were obtained for this study from the animal house of the faculty of veterinary medicine, Cairo University, Egypt. Mice were held in cages with complete access to filtered water and standard pellet. They were maintained under a temperature of 25 ± 2 °C as well as a 12 h light/dark cycle [34]. They were allowed to acclimatize for seven days. We followed the rules of using laboratory animals of the research ethics committee of the faculty of pharmacy, Tanta University, Egypt, with approval no. of TP/RE/05-22P-013.

#### 4.6.2. Experimental Model

First, mice were infected for two days via subcutaneous injection (SC) of *S. aureus* suspension (2.0 × 10^7^ CFU/mL). Then, mice were distributed in a random way into three groups (each group with ten mice). The groups were as follows: 1) group I was the un-treated infected group (or negative control), group II was the gentamicin treated infected group (or positive control, 2 mg/kg), and the third was the ZnO NPs treated infected group (5 mg/kg). The treatments were administered as intraperitoneal injection (IP) after 24 h from the second SC bacterial injection and continued for 20 days. Finally, all mice were anesthetized and euthanized via cervical dislocation. Blood was instantly obtained, and liver, as well as spleen tissue samples, were taken [13]. Additionally, a number of CFU/g were counted in the liver and spleen tissues [39]. 

#### 4.6.3. Histopathological Assessment

Liver and spleen tissue samples were taken from the mice and fixed using formalin (10%) for three days for histopathological examination [45]. The tissues were dehydrated, implanted in paraffin wax, and spliced into sections of three micrometres thickness. These sections were then stained with H&E as well as Masson’s trichrome stains [46,47]. A digital camera captured a photo of the stained tissues. 

#### 4.6.4. Liver Function Tests

Serum was utilized to measure the levels of ALT, AST, and albumin by the ab234579, ab263883, and ab108789 kits purchased from Abcam, USA. In addition, the total proteins (BioRad, Hercules, CA, USA) and bilirubin (MBS730053, MyBioSource, San Diego, CA, USA) were measured in all experimental groups.

#### 4.6.5. ELISA

Levels of IL-1β as well as IL-6 were assessed in the liver and spleen tissues using an ELISA kit (Abcam Co. Waltham, MA, USA). 

### 4.7. Statistical Analysis

Data of the current study were revealed as mean ± SD because all the assays were performed in triplicate. A one-way analysis of variance (ANOVA) assessed differences between the tested groups, followed by a post hoc test (Tukey). The change was regarded to be significant when *p* < 0.05. Additionally, the rate of survival of the rats was calculated using Kaplan–Meier survival curve. All these statistical calculations were performed using Prism version 8 (GraphPad Software, Inc., San Diego, CA, USA).

## 5. Conclusions

The present study highlighted the biogenic synthesis of ZnO NPs by the fungal filtrate of *A. niger* as a simple, cost-effective, and harmless method compared to chemical and physical methods. The prepared ZnO NPs were characterized by UV, FTIR, TEM, SEM, XRD, EDX, DSC, and DLS. Then, we investigated these nanoparticles’ in vitro and in vivo antibacterial potential. Interestingly, ZnO NPs exhibited antibacterial action against S. aureus clinical isolates. It also inhibited biofilm formation and resulted in a downregulation of the biofilm genes. Regarding their in vivo antibacterial activity, ZnO NPs caused a decrease in the congestion and fibrosis of the liver and spleen tissues. ZnO NPs could be considered as efficient nanocandidates that can be employed for multiple pharmaceutical and biological purposes in the future. 

## Figures and Tables

**Table 1:** Data of the molecular identification of *A. niger* endophytic fungus by 18S rRNA.

Accession Number	Identification	Highly Similarity Isolates	Highly Similarity Isolates Accession Number	Identity %
ON100821	*A. niger* isolate	*A. niger* CBS 554.65 18S rRNA gene, partial sequence	NG_065763.1	98.29

**Table 2:** Influence of ZnO NPs on the biofilm-forming ability of the tested *S. aureus.*

Biofilm Formation	No. of Isolates	
	Before Treatment (%)	After Treatment (%)
No biofilm formation	8 (33.34%)	13 (54.17%)
Weak	4 (16.67%)	6 (25%)
Moderate and strong	12 (50%)	5 (20.83%)

**Table 3:** Liver function tests in the experimental groups.

Groups	ALT (U/L)	AST (U/L)	Bilirubin (mg/dL)	Total Proteins (g/dL)	Albumin (g/dL)
Group I	98.2 ± 3.2	140.5 ± 4.3	0.74 ± 0.091	1.97 ± 0.12	1.3 ± 0.3
Group II	42.4 ± 1.3 *	68.4 ± 1.2 *	0.18 ± 0.002 *	7.1 ± 0.37 *	4.83 ± 0.28 *
Group III	41.0 ± 2.5 *	69.3 ± 2.7 *	0.19 ± 0.001 *	7.0 ± 0.29 *	4.91 ± 0.31 *

The symbol (*) represents a significant change (*p* < 0.05) in comparison with group I.

**Table 4:** Levels of IL-6 and IL-1β of the liver and spleen tissues of the different groups.

Tissues	Liver		Spleen	
**Groups**	**IL-6** **(pg/mg Tissues)**	**IL-1β** **(pg/mg Tissues)**	**IL-6** **(pg/mg Tissues)**	**IL-1**β **(pg/mg Tissues)**
Group I	222.1 ± 6.3	77.3 ± 3.4	210.4 ± 5.1	72.5 ± 5.1
Group II	101.1 ± 4.6 *	20.3 ± 3.7 *	99.9 ± 5.2 *	20.8 ± 3.2 *
Group III	99.8 ± 5.7 *	20.1 ± 4.3 *	100.1 ± 2.6 *	21.4 ± 2.6 *

The symbol (*) represents a significant decrease (*p* < 0.05) in comparison with group I.

## Data Availability

Data is contained within the article and supplementary material.

## Supplementary Materials

The following supporting information can be downloaded at: https://www.mdpi.com/article/10.3390/ph15091057/s1, Table S1. Sequences of the utilized primers; Table S2. MIC values of ZnO NPs against *S. aureus* clinical isolates.
