# Supplementary material for: Zinc Oxide Nanoparticles as Potential Delivery Carrier: Green Synthesis by Aspergillus niger Endophytic Fungus, Characterization, and In Vitro/In Vivo Antibacterial Activity
Source: Pharmaceuticals (Basel). 2022 Aug 26;15(9):1057. doi: 10.3390/ph15091057 (PMC9500724; doi:10.3390/ph15091057)
Supplement: Supplementary file 1 [file pharmaceuticals-15-01057-s001.zip › pharmaceuticals-1869544-supplementary.pdf]

Table S1. Sequences of the utilized primers

Gene	Primer sequence (5´ to3´)
*fnb*A	Fw: CAGTAGCTGAATTCCCATTTTCTTC<br>Rv: AAATTGGGAGCAGCATCAGT
*fnb*B	Fw: ACGCTCAAGGCGACGGCAAAG<br>Rv: ACCTTCTGCATGACCTTCTGCACC
*ebp*S	Fw: GCTGCGCCTCCAGCCAAACCT<br>Rv: GTGCAGCTGGTGCAATGGGTGT
*ica*C	Fw: TCTTGGGTATTTGCACGCAT<br>Rv: GCAATATCATGCCGACACCT
16srRNA	Fw: GGGACCCGCACAAGCGGTGG<br>Rv: GGGTTGCGCTCGTTGCGGGA

Table S2. MIC values of ZnO NPs against *S. aureus* clinical isolates.

Isolate code	MIC (μg/mL)	Isolate code	MIC (μg/mL)
S1	128	S13	128
S2	64	S14	64
S3	8	S15	64
S4	32	S16	32
S5	64	S17	32
S6	64	S18	16
S7	32	S19	8
S8	16	S20	128
S9	8	S21	64
S10	8	S22	16
S11	16	S23	16
S12	8	S24	8
